# Towards a theory of microbially-mediated invasion encompassing parasitism and mutualism

**DOI:** 10.1007/s10530-025-03711-4

**Published:** 2025-11-14

**Authors:** Maria M. Martignoni, Jimmy Garnier, Rebecca C. Tyson, Keith D. Harris, Oren Kolodny

**Affiliations:** 1 Department of Ecology, Evolution and Behavior, A. Silberman Institute of Life Sciences, Hebrew University of Jerusalem, Jerusalem, Israel; 2 CNRS, Université Savoie-Mont Blanc, Université Grenoble Alpes, Chambéry, France; 3 CMPS Department (Mathematics), University of British Columbia Okanagan, Kelowna, BC Canada

**Keywords:** Co-invasion, Symbiont spillover, Symbiont spillback, Microbially mediated, Microbe-mediated, Linked invasion, Mathematical model, Differential equation, Host-symbiont, Mutualism, Invasion, Microbial invasion, Plant invasion, Theoretical framework, Social microbiome

## Abstract

Biological invasions pose major ecological and economic threats, and extensive research has been dedicated to understanding and predicting their dynamics. Most studies focus on the biological invasion of single species, and only in recent years has it been realized that multi-species interactions that involve native and invasive host species and their microbial symbionts can play important roles in determining invasion outputs. A theoretical framework that treats these interactions and their impact is lacking. Here we offer such a framework and use it to explore possible dynamics that may emerge from the sharing of native and non-native symbionts among native and non-native host species. Thus, for example, invasive plants might benefit from native microbial communities in the soil, or might be particularly successful if they carry with them parasites to which competing native hosts are susceptible. On the other hand, invasion might be hindered by native parasites that spread from native to invasive individuals. The mathematical framework that we present in this study provides a new mechanistic, cohesive, and intuition-enhancing tool for theoretically exploring the ways by which the subtleties of host-microbe relationships can influence invasion dynamics. We identify multiple pathways through which microbes can facilitate (or prevent) host invasion, microbial invasion, and the invasion of both hosts and their co-introduced microbes. We disentangle invasion outcomes and suggest possible ecological dynamics that may be underexplored in current invasion biology literature. Our work sets the foundations for invasion theory that includes a community-level view of invasive and native hosts as well as their microbial symbionts.

**Supplementary Information:**

The online version contains supplementary material available at 10.1007/s10530-025-03711-4.

## Introduction

Biological invasions are often studied at the level of a single species. However, organisms live in symbiosis with a rich and diverse collection of microbial symbionts where both hosts and microbes are essential components of each other’s fitness and reproductive success (Fitzpatrick et al. 2020; Compant et al. 2019). Microbial communities can encompass mutualistic, commensal, and parasitic (or pathogenic) symbionts that benefit or harm the host to different extent. The fitness of these microbial symbionts, in turn, depends on their association with host partners. As such, the fates of hosts and symbionts are intrinsically linked, as are their chances of successfully establishing and persisting in a new environment. In some cases, invasion can be facilitated by interaction with native hosts or symbionts. For example, the formation of novel associations between invasive plants and pre-existing native mycorrhizal networks can facilitate the establishment and range expansion of introduced host populations (Dawkins and Esiobu 2016; Parepa et al. 2013; Moeller et al. 2015). Similarly, native hosts can inadvertently promote the spread of introduced symbionts (e.g., non-native pathogens), sometimes accelerating processes that contribute to their own displacement (Díez 2005; Wolfe and Pringle 2012; Dickie et al. 2016; Panzavolta et al. 2021; Santini et al. 2013; Grünwald et al. 2012; Schuchert et al. 2014). In other cases, host-symbiont interactions can provide resilience to native communities. For instance, a reduction in the abundance of mutualistic symbionts may limit host invasiveness (Zenni and Nuñez 2013; Catford et al. 2009), as can the transmission of pathogenic agents from native to invasive hosts (Mordecai 2013; Flory and Clay 2013).

Awareness is growing that host-symbiont interactions play an important role in both host and microbial invasions, and so are attempts at developing conceptual frameworks to understand the impact of these interactions on invasion outcomes (Dickie et al. 2017; Amsellem et al. 2017; Dunn and Hatcher 2015; Médoc et al. 2017; Mitchell et al. 2006; Nuñez et al. 2009; Dickie et al. 2017). From a theoretical standpoint, ecological population models have typically examined either parasite (White et al. 2018; Gubbins et al. 2000) or host invasion dynamics (Lewis et al. 2016), while rarely incorporating host-symbiont feedbacks that may critically shape invasion outcomes. Even in the few instances in which these feedbacks were considered (Bever et al. 1997; Yamauchi et al. 2011; Jack et al. 2017; Martignoni and Kolodny 2024; Kandlikar et al. 2019), studies mostly unilaterally discussed how diversity and fitness of the host population can be mediated by microbial communities and not vice-versa. Therefore, further research is needed to thoroughly evaluate how multi-species interactions involving native and invasive hosts and their microbial symbionts impact invasion dynamics.

Theoretical insights into the stability and resilience of mutualistic and parasitic communities against invasion have been provided by network theory. These approaches have mainly focused on relating community invasiveness to global structural features of the ecological community, such as nestedness, or connectivity (Campbell et al. 2012; Bastolla et al. 2009; Rohr et al. 2014; Vacher et al. 2010; Valdovinos 2019; Bascompte and Olesen 2015). In this framework, network structure can be informed by trait-based approaches that consider how trait differences and similarities between invaded and invading communities may influence interaction strengths (Minoarivelo and Hui 2016a; Hui et al. 2016; Minoarivelo and Hui 2016b; Schleuning et al. 2015; Runghen et al. 2021). While these methods provide valuable insights into the relationship between the architecture of species interactions and community stability, they often lack explicit representation of temporal dynamics. Additionally, theoretical results are largely based on Lotka–Volterra equations, which may lead to inaccurate predictions due to their unrealistic biological assumptions, such as linear positive effects of mutualistic interactions and unlimited growth (Holland 2015; Gibbs et al. 2024).

Here, we develop a differential equation model based on a consumer-resource framework, to capture the dynamic behavior of host and symbiont populations over time. Critically, our model incorporates interdependent fitness between hosts and symbionts, as well as density-dependent effects of interactions that go beyond the assumptions of classic Lotka-Volterra models. As such, our approach allows us to explore not only how network structure influences community outcomes, but also the precise population-level processes that drive invasion dynamics. Additionally, the consumer-resource framework we use allows for the explicit and separate characterization of both the benefits and costs of host-microbe interactions, each of which can depend on host and symbiont densities. This structure accommodates a continuum of host-symbiont associations, ranging from parasitic to mutualistic, within a unified modeling approach.

We use our framework to explore the various ways in which host-symbiont interactions can facilitate or hinder host invasion, symbiont invasion, or co-invasion, defined here as the simultaneous invasion of an introduced host and its associated symbionts. Specifically, we investigate the coupled population dynamics of a native and an invasive host species, along with their associated symbiont communities, which exert an average effect on their hosts. This simplification allows us to disentangle alternative mechanisms that can generate similar invasion patterns, and represents a first step toward a more mechanistic understanding of symbiont-mediated invasions.

## Model and methods

### Mathematical framework

To investigate the dynamics of host-symbiont communities we develop a consumer-resource model for mutualism, similar to that presented by Martignoni et al. (2020). We consider a native host population with biomass $p_n$ and its associated native symbiont community with biomass $m_n$. Hosts and symbionts interact by exchanging resources necessary for each other’s growth. For example, in the case of the mycorrhizal symbiosis, the host plant provides fixed carbon in the form of sugars (e.g., glucose and sucrose) to its associated mycorrhizal fungi, and receive necessary nutrients such as phosphorus, nitrogen, or water in return (Smith and Read 2010). The transfer of resources from hosts to symbionts, and from symbionts to host, is described by density-dependent $F_p$ and $F_m$ functions, detailed below. In our model, hosts are facultative mutualists, and capable of some growth in the absence of the symbionts (with intrinsic growth rate quantified by the parameter $r_p$), while symbionts are obligate mutualists and cannot grow in the absence of a host. We then extend this model to include interactions between an invasive host population (with biomass $p_i$) and its invasive symbiont community (with biomass $m_i$). Native symbionts may exchange resources with invasive hosts, and invasive symbionts may exchange resources with native hosts. Competition between native and invasive hosts ($c_p$ parameters) and between native and invasive symbionts ($c_m$ parameters), may reduce the abundance of hosts, symbionts, or both, e.g., due to competition for host colonization between symbionts (Engelmoer et al. 2014; Smith et al. 2018), or due to host competition for light or other external resources (Craine and Dybzinski 2013).

We obtain the following equations:

1a $$\begin{aligned} \dfrac{d p_n}{dt}&= r_{p_n} p_n + F_{p_n}(p_n,m_n,p_i,m_i)- c_{p_{in}} p_n p_i - \mu _{{p}_n} p_n^2\, , \end{aligned}$$

1b $$\begin{aligned} \dfrac{d m_n}{dt}&= F_{m_n}(p_n,m_n,p_i,m_i) - c_{m_{in}} m_n m_i - \mu _{{m}_n} m_n^2\, , \end{aligned}$$

1c $$\begin{aligned} \dfrac{d p_i}{dt}&= r_{p_i} p_i + F_{p_i}(p_n,m_n,p_i,m_i)- c_{p_{ni}} p_n p_i - \mu _{{p}_i} p_i^2\, , \end{aligned}$$

1d $$\begin{aligned} \dfrac{dm_i}{dt}&= F_{m_i}(p_n,m_n,p_i,m_i)- c_{m_{ni}} m_n m_i - \mu _{{m}_i} m_i^2 . \end{aligned}$$

Functions $F_{p_n}$, $F_{p_i}$, $F_{m_n}$, and $F_{m_i}$ determine the benefit and cost of the interaction and depend on host and symbiont biomass densities. We choose to use simple functional forms that capture the saturating nature of benefit transfer when plant and microbial biomasses differ substantially. These functions impose limitations on the exchange of benefits when one partner’s biomass far exceeds that of the other. Resource exchange is reduced in the presence of competitors. This formulation reflects a biologically realistic trade-off, where resource exchange depends on the relative capacity of interacting partners to give or receive.

Specifically, functions $F_{p_n}$ and $F_{p_i}$ (Eqs. (1a, c)) describe the amount of resources a host provides to its symbionts (i.e., host $\longrightarrow$ symbiont) is limited by the partner with the lower biomass in the interaction. When host biomass far exceeds symbiont biomass (adjusted by factor 1/*d*), the supply is capped by symbiont demand. Conversely, when symbiont biomass exceeds host biomass (adjusted by factor *d*), the supply is constrained by host capacity. Analogously, functions $F_{m_n}$ and $F_{m_i}$ (Eqs. (1b, d)) describe the amount of resources a symbiont provides to its hosts (i.e., symbiont $\longrightarrow$ host), and depends on the relative biomass of the partners. When host biomass exceeds symbiont biomass, the supply is limited by symbiont capacity to give, scaled by a factor of *d*. Conversely, when symbiont biomass exceeds host biomass, the exchange is constrained by host capacity to take, scaled by 1/*d*. We write:

2a $$\begin{aligned} F_{p_n} =&\underbrace{q_{hp_n} p_n \left( \dfrac{\varvec{\alpha }_{nn} m_n + \varvec{\alpha }_{in} m_i}{p_n/d+p_i/d+m_n+m_i}\right) }_{\mathrm {supply~from~m_n~and~m_i~to~p_n}}- \underbrace{q_{cp_n} p_n \left( \dfrac{\varvec{\beta }_{nn} m_n + \varvec{\beta }_{ni} m_i}{p_n+p_i+dm_n+dm_i} \right) }_{\mathrm {supply~to~m_n~and~m_i~by~p_n}} , \end{aligned}$$

2b $$\begin{aligned} F_{m_n} =&\underbrace{q_{cm_n} m_n \left( \dfrac{ \varvec{\beta }_{nn} p_n + \varvec{\beta }_{in} p_i}{p_n + p_i+dm_n+dm_i} \right) }_{\mathrm {supply~from~p_n~and~p_i~to~m_n}} - \underbrace{q_{hm_n} m_n \left( \dfrac{\varvec{\alpha }_{nn} p_n + \varvec{\alpha }_{ni} p_i }{p_n/d + p_i/d + m_n + m_i}\right) }_{\mathrm {supply~to~p_n~and~p_i~by~m_n}} , \end{aligned}$$

2c $$\begin{aligned} F_{p_i} =&\underbrace{q_{hp_i} p_i \left( \dfrac{\varvec{\alpha }_{ii} m_i + \varvec{\alpha }_{ni} m_n}{p_n/d+p_i/d+m_n+m_i}\right) }_{\mathrm {supply~from~m_i~and~m_n~to~p_i}}- \underbrace{q_{cp_i} p_i \left( \dfrac{\varvec{\beta }_{ii} m_i + \varvec{\beta }_{in} m_n}{p_n+p_i+dm_n+dm_i} \right) }_{\mathrm {supply~to~m_i~and~m_n~by~p_i}} , \end{aligned}$$

2d $$\begin{aligned} F_{m_i} =&\underbrace{q_{cm_i} m_i \left( \dfrac{\varvec{\beta }_{ii} p_i + \varvec{\beta }_{ni} p_n}{p_n + p_i+dm_n+dm_i} \right) }_{\mathrm {supply~from~p_i~and~p_n~to~m_i}} - \underbrace{q_{hm_i} m_i \left( \dfrac{\varvec{\alpha }_{ii} p_i + \varvec{\alpha }_{in} p_n }{p_n/d + p_i/d + m_n + m_i}\right) }_{\mathrm {supply~to~p_i~and~p_n~by~m_i}}. \end{aligned}$$

**Fig. 1:**
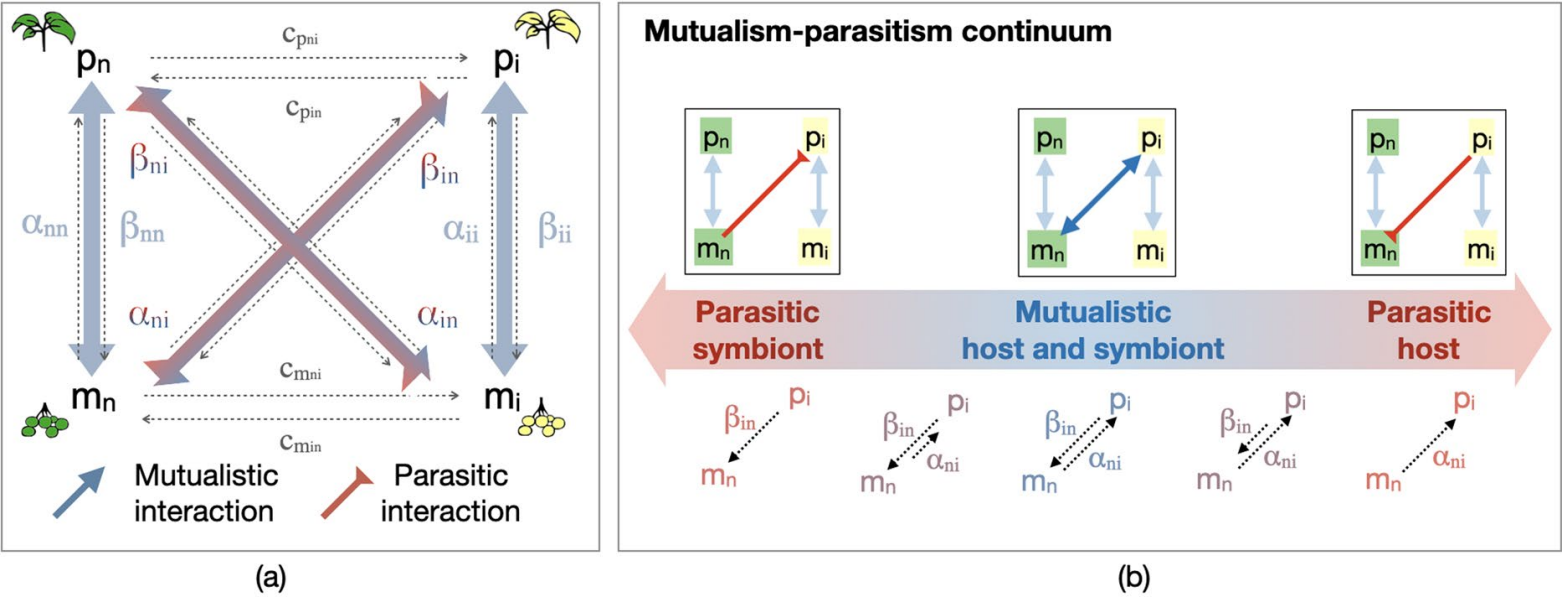
**a** Schematic representation of the model of Eq. (1). A native microbial community $m_n$ interacts with a host population $p_n$. Resource exchange between hosts and symbionts is quantified by parameters $\alpha _{nn}$ (symbionts to hosts) and $\beta _{nn}$ (hosts to symbionts). Similarly, a population of invasive hosts $p_i$ exchanges resources with its associated invasive symbiont community $m_i$ (parameters $\alpha _{ii}$ and $\beta _{ii}$). Depending on the scenario considered, invasive hosts may also exchange resources with native symbionts (parameters $\alpha _{ni}$ and $\beta _{in}$), and invasive symbionts may exchange resources with native hosts (parameters $\alpha _{in}$, and $\beta _{ni}$). The red to blue colour gradient indicates the degree to which host-symbiont interactions are, respectively, parasitic or mutualistic for hosts and symbionts, as shown in (**b**). Additionally, native and invasive hosts compete with each other, with competition strength quantified by parameters $c_{p_{in}}$ and $c_{p_{ni}}$. Similarly, there is competition between native and invasive symbionts (parameters $c_{m_{in}}$ and $c_{m_{ni}}$)

A schematic representation of our model is provided in Fig. 1a. A table with a brief description of model parameters is provided in SI A.

The transfer of resources from symbionts to hosts and from hosts to symbionts is quantified by the $\alpha _{jk}$ and $\beta _{jk}$ parameters respectively, with subindex *j* representing the supplying species (*n* for native, or *i* for invasive), and subindex *k* representing the receiving species. These parameters may represent particular traits of the receiving and supplying species that quantify their resource exchange capacity. For instance, microbes that provide lots of phosphorus to host plants and take lots of carbon from host plants are represented by large $\alpha$ and $\beta$ parameters. The efficiency of native and invasive species in converting the resources they receive or supply into biomass is captured by the *q* parameters, while all $\mu$ parameters represent the rates at which resources are allocated to the maintenance of existing biomass.

To explore the effect of host-symbiont associations on invasion dynamics, we consider that parameters $\alpha _{in}$, $\alpha _{ni}$, $\beta _{in}$ and $\beta _{ni}$ can be zero or positive, depending on whether or not resource exchange between invasive symbionts/hosts and native hosts/symbionts is occurring. If invasive hosts exchange nutrients with native symbionts, parameters $\alpha _{ni}$ and $\beta _{in}$ will assume positive values. The time-dependent relationship between how much a host receives from and gives to its associated symbionts (which depends on the $\alpha$ and $\beta$ parameters, respectively) determines whether a host-symbiont relationship is mutualistic or parasitic for the host or the symbiont population (see Fig. 1b). In the simplest case, the relationship is parasitic for the host population if $\alpha \ll \beta$, and parasitic for the symbiont community if $\beta \ll \alpha$, while the interaction is mutualistic for both hosts and symbionts, for $\alpha \simeq \beta$. A detailed explanation of the quantitative criteria used to understand whether the exchange is mutualistic or parasitic is provided below in the “Model analysis and scenarios of interest” section.

### Model analysis and scenarios of interest

In our model, microbial contribution to host growth and host contribution to microbial growth vary along a continuum ranging from ‘parasitic’ to ‘mutualistic’ (see Fig. 1b). A certain host-symbiont association is defined as ‘mutualistic’ if the interaction increases the growth rate of both hosts and symbionts. An association is considered ‘parasitic’ if the growth rate of either hosts or symbionts, is decreased by the interaction.

Specifically, we examine a 2-equation version of the model in Eq. (1) for a host population *p* (either $p_n$ or $p_i$) interacting with a microbial community *m* (either $m_n$ or $m_i$). We obtain:

3 $$\begin{aligned} {\left\{ \begin{array}{ll} \dfrac{dp}{dt} & = r_{p} p + \dfrac{p m }{\dfrac{p}{d} + m} \underbrace{\left( q_{hp} \dfrac{\alpha }{d} - q_{cp} \beta \right) }_{:= Q_p} - \mu _{p} p^2 \, , \\ \dfrac{dm }{dt} & = \dfrac{p m }{\dfrac{p}{d} + m} \underbrace{ \left( q_{cm} \beta - q_{hm} \dfrac{\alpha }{d} \right) }_{:= Q_m} - \mu _{m} m^2 \, . \end{array}\right. } \end{aligned}$$

The association between *p* and *m* is: mutualistic for $Q_p,Q_m>0$; parasitic for the host population if $Q_p<0$ and $Q_m>0$; and parasitic for the microbial community if $Q_p>0$ and $Q_m<0$. Note that the values of $Q_p$ and $Q_m$ depend on parameters $\alpha$ and $\beta$, which characterise the quality of the resource exchange between *p* and *m*.

**Fig. 2:**
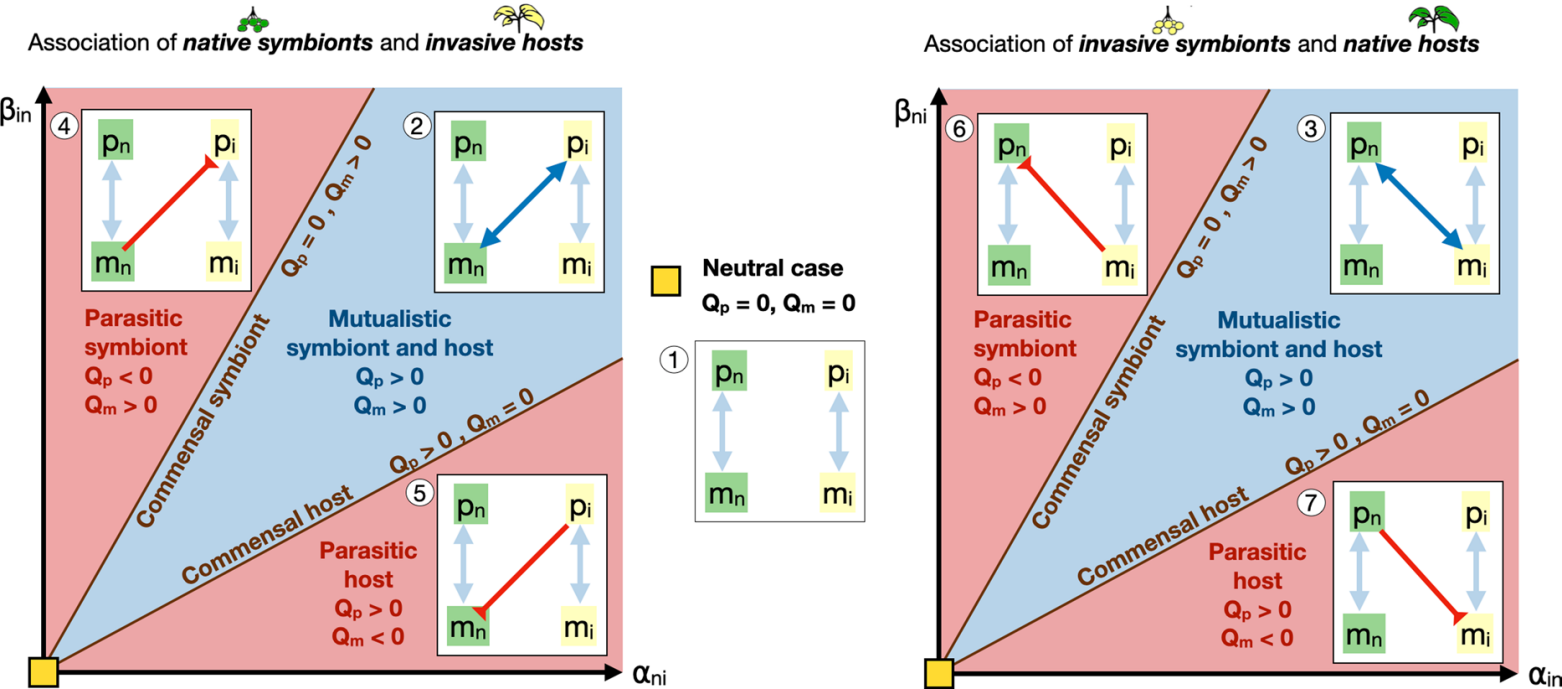
Novel host-symbiont interactions between native microbes and invasive hosts (left plot) or between native hosts and invasive microbes (right plot) can be parasitic or mutualistic, depending on the value of parameters $\alpha _{in}$, $\alpha _{ni}$, $\beta _{ni}$ and $\beta _{in}$, quantifying the resource exchange capacity of hosts and symbionts (see also Fig. 1). We characterize 7 different parameter regions, corresponding to scenarios 1–7, discussed in the manuscript. Left plot: Scenarios 2, 4, and 7; Middle plot: Scenario 1; Right plot: Scenarios 3, 6, and 7. The mathematical definition of the thresholds $Q_p$ and $Q_m$ are provided in Eq. (3) and Eqs.  (4) and (5)

We restrict our attention to the case where the association between native hosts and native symbionts (i.e., between $m_n$ and $p_n$) and between invasive hosts and invasive symbionts (i.e., between $m_i$ and $p_i$) is mutualistic. We then consider the possible outcomes when the associations between native symbionts $m_n$ and invasive hosts $p_i$ (Fig. 2, left) and between invasive symbionts $m_i$ and native hosts $p_n$ (Fig. 2, right) can be mutualistic or parasitic.

For instance, the association between native host $p_n$ and invasive microbial symbiont $m_i$ is mutualistic as long as

4 $$\begin{aligned} \begin{array}{cc} Q_p> 0~~~\Longleftrightarrow \alpha _{in}> \dfrac{q_{cp_n}}{q_{hp_n}} \beta _{ni} d\,~~~ \& ~~~ Q_m> 0~~~\Longleftrightarrow \beta _{ni} > \dfrac{q_{hm_i}}{q_{cm_i}} \dfrac{\alpha _{in}}{ d}. \end{array} \end{aligned}$$

Conversely, the interaction is parasitic for either the host or the symbiont if $Q_p<0$ or $Q_m<0$. Analogously, association between invasive host $p_i$ and native microbial symbiont $m_n$ is mutualistic for both host and symbiont if

5 $$\begin{aligned} \begin{array}{cc} \alpha _{ni}> \dfrac{q_{cp_i}}{q_{hp_i}} \beta _{in} d\,~~~ \& ~~~ \beta _{in} > \dfrac{q_{hm_n}}{q_{cm_n}} \dfrac{\alpha _{ni}}{ d}, \end{array} \end{aligned}$$

(see also SI B.2 for more details).

The straight lines defined by Eqs. (4) and (5), in the $(\alpha ,\beta )$-parameter space, mark the borders between the different regions in Fig. 2, i.e., parasitic symbiont, mutualistic symbiont and host, and parasitic host. In these regions, interactions between an invasive host population $p_i$ and a native symbiont community $m_n$ is considered (left plot), or interactions between a native host population $p_n$ and an invasive symbiont community $m_i$ is considered (right plot). Equality in either terms of Eqs. (4) and (5) represents cases in which either hosts or symbionts are commensals.

We obtain seven different scenarios, which are identified in Fig. 2: $\textcircled {1}$ Invasive and native hosts do not share symbionts; a mutualistic association is observed between $\textcircled {2}$ invasive hosts and a native symbionts, or $\textcircled {3}$ native hosts and invasive symbionts; a parasitic association is observed, in which native/invasive symbionts exploit invasive/native hosts ($\textcircled {4}$ and $\textcircled {6}$), or invasive/native hosts exploit native/invasive symbionts ($\textcircled {5}$ and $\textcircled {7}$). The mathematical analysis of these scenarios and their biological significance will be discussed in the “Scenarios of interest” section. Combinations of these scenarios will be considered in the “Combined scenarios”  section.

## Results

### Overview of model analysis

In the following, we discuss the interesting dynamics emerging from scenarios $\textcircled {1}$–$\textcircled {7}$, which shed light on several ways in which host-symbiont interactions can lead to invasion of hosts, symbionts, or both. Note that the directionality of the interactions is important, i.e., we distinguish between the effect of hosts on symbionts, and the effect of symbionts on hosts. The outcome may be the same in separate scenarios, but the mechanisms leading to these outcomes differ.

A detailed mathematical analysis of these scenarios is presented in the SI B-E, and includes: the analysis of the one host—one symbiont system (section B), which is crucial for defining the parasitic/mutualistic interactions between a host population and its symbiont community; the analysis of the one host—two symbiont system (section C), which is important to understand the dynamics of a host population (native or invasive) associating with both native and invasive symbionts; the analysis of the one symbiont—two hosts system (section D), providing insights on the dynamics of a symbiont community (native or invasive) associating with native and invasive hosts; and the analysis of the two hosts—two symbionts system analyzed according to the scenarios described in $\textcircled {1}$–$\textcircled {7}$ (section E). We refer to invasion as a situation in which the successful establishment and persistence of invasive hosts, symbionts or both is possible. Exploration of alternative scenarios not presented in this paper, such as specific scenarios corresponding to different parameter combinations, can be conducted through numerical simulations of Eq. (1). For precisely this purpose, a user-friendly version of the code is made publicly available on the modelRxiv platform (Harris et al. 2022) (https://modelrxiv.org/model/YfndNX).

### Scenarios of interest

$\textcircled {1}$ ***Native and invasive species do not share symbionts nor hosts.***

When neither symbionts nor hosts are shared between native and invasive species, and competition within hosts and between symbionts is strong, competitive exclusion occurs, with either the native or invasive host-symbiont community outcompeting the other. This exclusion can result from selection driven by trait differences (represented in the model through differences in model parameters $\alpha$, $\beta$, *q*, *c*, $\mu$, or $r_p$, which can be explored numerically), or through differences in initial abundance, i.e., propagule density. When trait-based selection occurs, the host-symbiont association that confers the highest fitness to hosts or symbionts prevails. Notably, in this case more mutualistic associations tend to provide higher fitness and thus greater resilience against invasion. Differences in initial abundance can also give one community an advantage (see also Fig. E.1$\textcircled {1}$). Indeed, greater initial abundance of symbionts or hosts enhances the growth rate of their partners, creating a positive feedback loop that favors the community with the larger starting population.

**Fig. 3:**
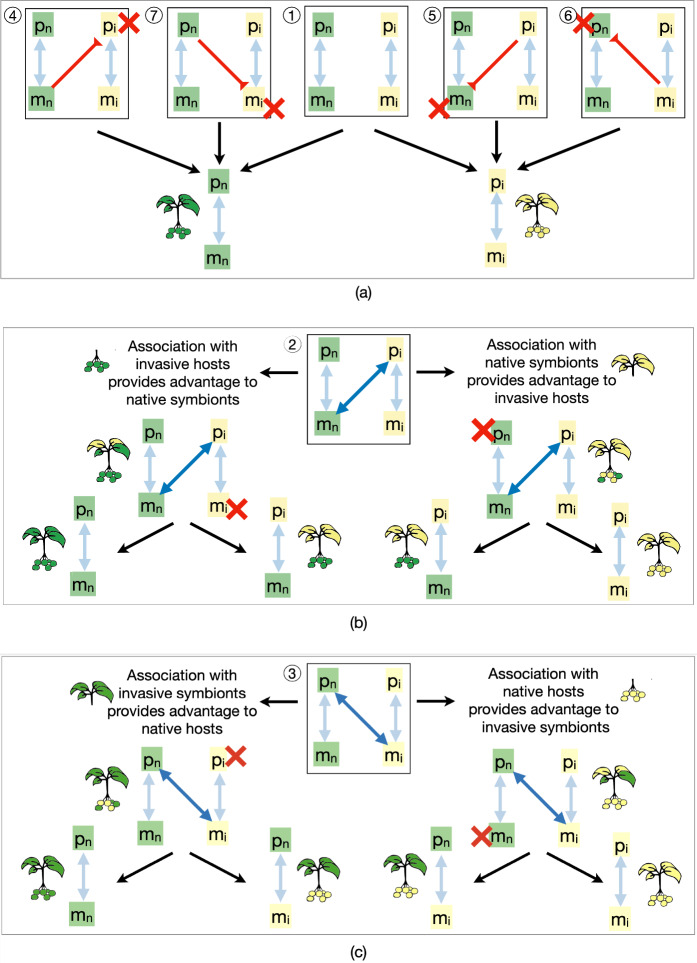
Possible dynamics of invasion arising when native species form novel host-symbiont associations with invasive species, for each of the scenarios described in Fig. 2. **a** Co-invasion can be facilitated (scenarios 5 and 6) or prevented (scenarios 4 and 7) by the formation of parasitic associations between native and invasive species. Association of **b** native symbionts with invasive hosts (scenario 2) or (**c**) invasive symbionts with native hosts (scenario 3) can confer competitive advantages to hosts or symbionts, where outcomes vary depending on whether hosts or microbes competitively exclude one another first

$\textcircled {2}$ ***The association between native symbionts and invasive hosts is mutualistic***

**Fig. 4:**
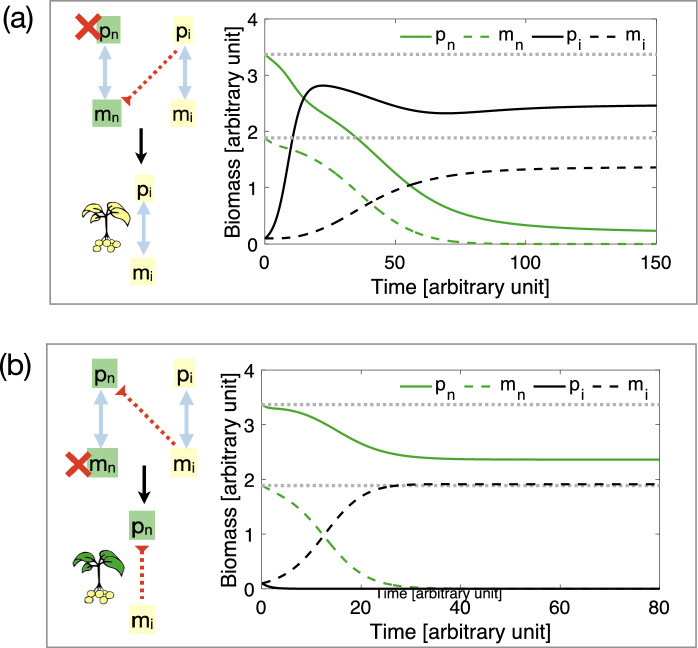
**a** Possible dynamics of co-invasion when invasive hosts act as low-quality mutualists, exploiting native symbionts at the indirect expense of native hosts. As a result, the post-invasion community may exhibit lower total biomass than the displaced native community (compare black curves and dotted grey horizontal lines). **b** Possible dynamics of microbial invasion when native hosts associate with invasive symbionts that are low-quality mutualists. In this case, invasive symbionts persist in the environment alongside native hosts, resulting in a community with lower total biomass than the original one. Interactive simulation of these and related scenarios can be done through the modelRxiv platform (https://modelrxiv.org/model/YfndNX)

- *Association with native symbionts provides advantage to invasive hosts: * Association with native symbionts can increase the fitness of invasive hosts, and provide them with a competitive advantage that can lead to competitive exclusion of native hosts (Fig. 3b, right pathways, and SI E and Fig. E.1$\textcircled {2}$ for mathematical insights). Subsequently, invasive symbionts may outcompete native symbionts, e.g., if native symbionts are weakened by the absence of native hosts or if invasive symbionts are empowered by an increase in invasive hosts (Fig. 3a, far right pathway, leading to association between $p_i$ and $m_i$). Alternatively, invasive symbionts may be outcompeted by native symbionts (Fig. 3a, center right pathway, leading to association between $p_i$ and $m_n$).
  A variation of this scenario, where invasive hosts act as low-quality mutualists, is discussed in $\textcircled {5}$ and in Fig. 4a. In this case, native symbionts receive less benefit from invasive hosts compared to what they receive from native hosts, yet they provide similar benefits to both native and invasive hosts. As a result, invasive hosts may grow rapidly by exploiting the native microbial community and, indirectly, the native hosts. Thus, native symbionts and hosts may be disadvantaged by the presence of invasive hosts that gain resources from native symbionts while providing little in return. If this dynamic leads to the displacement of native hosts and symbionts, invasive hosts will lose their low-cost resource advantage, and the resulting invasive host-symbiont community will have lower biomass than the original native community.
  Note that in the scenario where native and invasive species do not share symbionts (i.e., scenario $\textcircled {1}$), less mutualistic host-symbiont associations are expected to have lower fitness compared to more mutualistic ones and are therefore unlikely to invade. However, when novel host-symbiont associations are possible, co-invasion can occur through the exploitation of existing associations, with invasive hosts indirectly benefiting by receiving resources from native symbionts at low cost, thereby exploiting native hosts.
- *Association with invasive hosts provides advantage to native symbionts:* Mutualistic association with invasive hosts can also provide a competitive advantage to native symbionts, that then outcompete invasive symbionts (Fig. 3b, left pathway). No invasion occurs if invasive hosts suffer from the disruption of invasive host-symbiont associations which causes them to be outcompeted by native hosts (Fig. 3b, far left pathway, leading to association between $p_n$ and $m_n$). However, if invasive symbionts are low-quality mutualists (or parasites) to their own invasive hosts, their exclusion can provide a competitive advantage to invasive hosts, and cause the subsequent exclusion of native hosts. In this case, we observe the formation of novel associations between invasive hosts and native symbionts (Fig. 3b, certre left pathway, leading to association between $p_i$ and $m_n$). Note that this scenario of microbial invasion through host replacement may be more likely to occur, as it can be observed through two different pathways, namely, the center left and right pathways in Fig. 3b (see also SI E and Fig. E.1$\textcircled {2}$ for details on the dynamics).

$\textcircled {3}$ ***The association between native hosts and invasive symbionts is mutualistic***

- *Association with native hosts provides advantage to invasive symbionts:* If association with native hosts provides a competitive advantage to invasive symbionts, they may subsequently outcompete native symbionts (Fig. 3c, right pathways, and SI E and Fig. E.1$\textcircled {3}$ for details). The disruption of the association between native hosts and their symbionts may weaken native hosts, and lead to the establishment of invasive host-symbiont associations (Fig. 3c, far right pathway, leading to association between $p_i$ and $m_i$). Alternatively, microbial invasion may be observed if invasive hosts are outcompeted by native hosts, but invasive symbionts persist in association with native hosts (Fig. 3b certre right pathway, leading to association between $p_n$ and $m_i$).
  A variation of the latter scenario is the situation in which invasive symbionts are not completely harmful to native hosts, but act as low-quality mutualists. In this case, replacing native symbionts with invasive ones may result in a reduction of native host biomass (Fig. 4b). Consequently, invasive symbionts can persist in the environment and continue to negatively impact ecosystem functionality long after their invasive hosts have disappeared.
- *Association with invasive symbionts provides advantage to native hosts:* Association with invasive symbionts can also provide an advantage to native hosts, which then outcompete invasive hosts (Fig. 3c, left pathway and Fig. E.1$\textcircled {3}$). If invasive symbionts are strong competitors, they may outcompete native symbionts, leading to the formation of novel associations between invasive symbionts and native hosts, thereby facilitating microbial invasion (Fig. 3c certre left pathway, leading to association between $p_n$ and $m_i$). This scenario may be more likely to occur, as it can be observed through two different pathways. No invasion occurs if invasive symbionts suffer from the absence of invasive hosts and are outcompeted by native symbionts (Fig. 3b far left pathway, leading to associations between $p_n$ and $m_n$).

$\textcircled {4}$ ***Native symbionts are parasitic to invasive hosts.***

The association of native symbionts with invasive hosts can provide biotic resistance to native host-symbiont associations (Fig. 3a, pathway $\textcircled {4}$ and SI E and Fig. E.1$\textcircled {4}$). This can occur, for example, if parasites that are only slightly harmful to native hosts, e.g., because they have co-evolved with them, cause significant fitness reduction or death of invasive hosts. The death of invasive hosts would then be followed by the death of their symbionts. Poor adaptation of invasive species to native symbionts can result in the exploitation of invasive hosts by native symbionts. For example, this can occur if native symbionts obtain resources from invasive hosts at low cost while weakening their hosts (see also Fig. D.2 for details).

$\textcircled {5}$ ***Invasive hosts are parasitic to native symbionts.***

If invasive hosts directly harm microbial communities (e.g., by taking resources from native symbionts without reciprocation) the resulting interaction can weaken native symbionts, leading to their displacement and, subsequently, the displacement of native hosts (Fig. 3a, pathway $\textcircled {5}$ and SI E and Fig. E.1$\textcircled {5}$). This scenario could arise if the signaling dynamics between host and symbiont have not co-evolved. As a result, the host might exploit the symbiont to its own advantage, against the symbiont’s evolutionary interests. Due to the lack of prior interactions, the symbiont may fail to detect or adapt to this exploitation, creating a novel type of eco-evolutionary trap (Ferriere and Legendre 2013). If invasive hosts are not entirely harmful to native symbionts but act as low-quality mutualists, their association with native symbionts can facilitate the exploitation of existing native host-symbiont relationships to promote invasion, as discussed in $\textcircled {2}$ and Fig. 4a (see also Fig. D.2 for details).

$\textcircled {6}$ ***Invasive symbionts are parasitic to native hosts.***

Co-invasion may be facilitated when invasive symbionts harm native species, weakening native hosts and leading to their competitive exclusion along with the displacement of their symbionts (see Fig. 3a, pathway $\textcircled {6}$ and SI E and Fig. E.1$\textcircled {6}$). This situation can occur, for example, when pathogens that cause disease in native hosts are co-introduced with invasive hosts. A similar dynamic arises if invasive symbionts are low-quality mutualists that weaken native hosts, leading to their competitive exclusion by invasive hosts (see Fig. C.1).

$\textcircled {7}$ ***Native hosts are parasitic to invasive symbionts.***

Interaction of native hosts with invasive symbionts can provide resilience against invasion if native hosts are harmful to invasive symbionts (Fig. 3a, pathway $\textcircled {7}$ and SI E and Fig. E.1$\textcircled {7}$). Here, we consider that a native host can take advantage of invasive symbiont to the symbiont’s detriment, preventing the establishment of invasive symbionts and their hosts (see also Fig. C.1).

### Combined scenarios

**Fig. 5:**
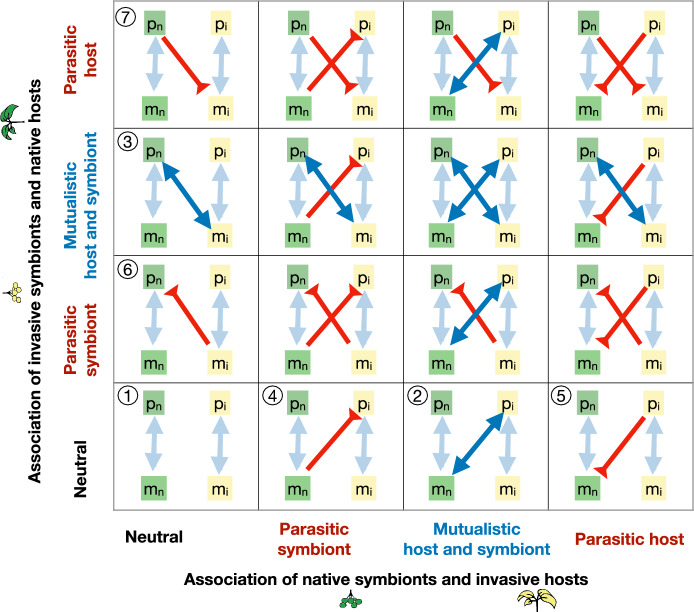
Schematic representation of interaction scenarios that can be explored within our framework. The seven numbered circles correspond to the scenarios discussed in the Results section. Additional scenarios can be interpreted as combinations of these seven. Interactive simulation of these and related scenarios, with our default parameters or user-defined values, can be done through the modelRxiv platform (https://modelrxiv.org/model/YfndNX)

In the previous section, we discussed how novel host-symbiont associations can affect invasion dynamics. While those scenarios considered the influence of native species on invasive ones, or vice versa, it is also possible for both native and invasive species to form novel associations simultaneously (see Fig. 5). For example, invasive hosts may simultaneously acquire mutualistic native symbionts (thereby strengthening themselves) and transmit parasitic symbionts to native hosts (thereby weakening them) (see Fig. 5, third row and column). This dual process can increase the competitive ability of invasive hosts while reducing that of native hosts, ultimately facilitating host invasion. Likewise, microbial invasion is more likely to occur when invasive symbionts are strengthened through novel associations with native hosts, while native symbionts are simultaneously harmed by invasive hosts (Fig. 5, second row, fourth column, counting from the top left).

## Discussion

### Host-symbiont interactions increase invasion risk

A growing number of empirical studies, especially on plant-fungal associations, show that invasion can be facilitated when invasive hosts form novel mutualistic relationships with native symbionts, thereby enhancing their competitive ability (Callaway et al. 2004; Tedersoo et al. 2007; Moeller et al. 2015; Shipunov et al. 2008; Keet et al. 2021). This rapid formation of new biotic interactions, often termed ‘ecological fitting’, has been observed even when invasive hosts and native symbionts share little co-evolutionary history (Le Roux et al. 2017). Strong evidence also shows that pathogens co-introduced with invasive hosts can weaken native host populations, thereby facilitating their competitive exclusion by invaders in a process often referred to as ‘disease-mediated invasion’ (Anderson et al. 2004; Parker and Gilbert 2004; Desprez-Loustau et al. 2007; Santini et al. 2013; Carnegie et al. 2016). We highlight that these dynamics extend beyond straightforward pathogen or mutualist sharing: differences in the mutualistic quality of introduced symbionts, due for example to a lack of co-evolution or specialization (Le Roux et al. 2017), may also influence invasion outcomes. For instance, introduced symbionts that are slightly less mutualistic than those in native communities may lead to a decrease in native host biomass (Bever 2002) (e.g., see our Fig. 4b). Similarly, acquiring native symbionts that are more mutualistic than those in the original invasive community can facilitate host invasion (see Fig. 4a). Though such changes may not directly cause community extinction as with pathogenic microbes, they can shift competitive balances and alter invasion dynamics (Levine et al. 2004).

In some cases, the formation of novel host-symbiont interactions may alter total community biomass, leading to long-term effects on ecosystem functionality (Nunez and Dickie 2014; Lovett et al. 2006; Dickie et al. 2011; Mitchell and Power 2003; Cobb and Rizzo 2016; Preston et al. 2016; Ehrenfeld 2010). While reductions in community biomass due to pathogen spread are well-documented (Lovett et al. 2006; Mitchell and Power 2003; Cobb and Rizzo 2016; Preston et al. 2016), here we present alternative mechanisms by which invasion can drive biomass decline (Billick and Case 1994; Mayfield and Stouffer 2017). If invasive hosts offer lower rewards than native hosts (Hoffman and Mitchell 1986; Mummey and Rillig 2006; Hausmann and Hawkes 2009; Vogelsang and Bever 2009), their association with native symbionts results in direct exploitation of symbiont resources. Moreover, native hosts that previously invested in maintaining large mutualistic symbiont communities experience indirect exploitation, as they can no longer fully benefit from these associations. Consequently, the proliferation of invasive hosts, supported by pre-existing native host-symbiont networks, can lead to declines in both native symbiont populations and their native hosts (e.g., see Fig. 4a).

In addition to highlighting the possible dynamics along the host-symbiont mutualism-parasitism continuum, we emphasize the importance of considering invasion dynamics at the community level, incorporating not only host-symbiont interactions but also host–host and symbiont–symbiont interactions. Focusing solely on host-symbiont interactions may lead to the misconception that only the host-symbiont community conferring the highest fitness to both hosts and symbionts can co-invade and displace native communities, as has been observed in some cases (Dickie et al. 2010; Nunez and Dickie 2014; Hayward et al. 2015). However, when considering that symbiont disruption and exchange between native and invasive species can occur (Dickie et al. 2017; Catford et al. 2009; Mitchell et al. 2006), co-invasion by a less fit host-symbiont community may take place (Fig. 4a). Thus, understanding the full range of possible outcomes following the introduction of a new species requires embracing a community perspective that accounts for interactions among multiple hosts and symbionts (Dickie et al. 2017; Fahey and Flory 2022).

### Host-symbiont interactions increase community resilience

Less studied than the role of host-symbiont associations in invasion dynamics is their role in providing resilience to native communities (Van der Putten et al. 2010; Zenni and Nuñez 2013; Levine et al. 2004). Despite its importance for community assembly (Wu et al. 2024), invasion failure remains poorly understood in practice (Diez et al. 2009; Zenni and Nuñez 2013). While novel host-symbiont associations may be crucial to explaining invasion success, they can also play a role in conferring resistance to invasion. Indeed, mechanisms such as symbiont disruption or lack of introduction (Le Roux et al. 2017), along with changes in community composition and emergent properties following species introduction, may alter resource exchange dynamics between hosts and symbionts and potentially provide resistance to invasion (Levine et al. 2004; Dinoor and Eshed 1984; Beckstead and Parker 2003; Knevel et al. 2004).

A clear example of host-symbiont associations driving invasion failure is the transmission of native pathogens to invasive plants (Hood et al. 2008; Piou et al. 2002). Such novel associations can provide biotic resistance against invaders (Mordecai 2013; Flory and Clay 2013; Prevéy and Seastedt 2015). Other, more complex dynamics of biotic resistance occur when native symbionts (having co-evolved with native hosts) are highly mutualistic to natives but provide low-quality mutualism to invasive hosts (Bunn et al. 2015; Moora et al. 2011; Le Roux et al. 2017). We show that association with such low-quality mutualistic symbionts can harm invasive hosts and facilitate their competitive exclusion by native hosts.

Finally, less competitive host-symbiont pairs may resist invasion by forming associations with mutualistic invasive symbionts (Mordecai 2013; Flory and Clay 2013). Reports of invasive symbiont spread in native habitats are numerous (Dickie et al. 2016; Wolfe and Pringle 2012; Berch et al. 2017; Mallon et al. 2015; Hart et al. 2017; Golan et al. 2024), yet the ecological consequences of these novel associations remain unclear. Our framework can guide empirical research aimed at understanding when such associations provide natives with biotic resistance to host invasion and when they may instead pose a risk by facilitating symbiont invasion.

### Framework limitations and possible extensions

Our framework allows for the mechanistic exploration of the interactions between microbes and their hosts, and can produce a rich variety of theoretical scenarios. It offers a solid starting point for new mathematical exploration by incorporating different levels of interactions (i.e., among hosts, among symbionts, and between hosts and symbionts). It also includes ecologically plausible functional responses (see Eqs. (2)) and allows for asymmetries in mutualistic dependence, such as differences between obligate and facultative relationships. As such, our work should be viewed as a foundational step, laying the theoretical groundwork for deeper investigations involving multi-species interactions and evolutionary processes.

A natural extension of this framework includes accounting for associations between multiple hosts and symbionts (see SI F). For example, one could consider a microbial community composed of multiple microbial strains in which invasive hosts preferentially support certain microbial strains over others (Callaway et al. 2001; Bever 2002; Kohout et al. 2011)). This setup could help us investigate the effect of higher-order interactions on coexistence and diversity of large host-symbiont communities. Other possible extensions include the evolution of host-symbiont associations, such as the evolution of host adaptation to pathogens (Thrall et al. 2002), or ‘parasite-spillback’ mechanisms (Flory and Clay 2013; Strauss et al. 2012; Kelly et al. 2009; Mangla et al. 2008; Day et al. 2016). These cases include invasive hosts forming associations with native pathogens that proliferate on the invasive hosts, ultimately resulting in increased infection pressure on native hosts.

Propagule pressure, defined as the total number of individuals introduced into a new environment and the frequency of these introductions, is also a major determinant of invasion success (Stringham and Lockwood 2021). Our model explicitly captures propagule densities through the initial biomass of invasive hosts and symbionts. Extending our framework to incorporate repeated introductions or continuous propagule input could offer additional valuable insights into how introduction frequency influences invasion dynamics and long-term establishment (Hui et al. 2021). Finally, our deterministic model cannot fully capture the important roles of demographic stochasticity and environmental fluctuations, which are especially influential during the early stages of invasion (see Cuddington and Hastings (2016)).

### Applicability of the framework to real-world systems

Assessing the relevance of the modeled scenarios to real-world systems requires a case-by-case approach that considers the specificity of host-symbiont interactions and their context-dependent effects on host fitness. Our terminology and structure align most closely with symbioses in which microbes are primarily external, such as many plant-microbial associations, because these systems have been well studied in the context of symbiont-mediated invasion (Dickie et al. 2017; Bever et al. 2010). The framework can also be applied to internal or partially internal symbionts, including gut microbiomes or coral symbionts (Pettay et al. 2015; Chiarello et al. 2022; Goedknegt et al. 2017), but doing so may require more caution. Internal symbionts are subject to spatial constraints, host regulation, and specialized transmission dynamics, which can modify interaction strengths and invasion outcomes compared to external associations.

The strength of our model lies less in precise quantitative prediction than in its ability to illuminate simplified yet qualitatively distinct scenarios, fostering intuition and mechanistic understanding of symbiont-mediated invasion dynamics. Nonetheless, quantitative insights remain possible: parameterizing the model with ecologically grounded values allows numerical simulations (e.g., via the modelRxiv platform; Harris et al. (2022)) to probe potential invasion trajectories and identify outcomes most likely under specific ecological conditions.

While the model contains many parameters, its essential dynamics can be fully understood through a reduced set. As explained in the “Model analysis and scenarios of interest” section and illustrated in Fig. 2, the parameters $Q_p$, and $Q_m$ (defined in Eq. (3)), govern the main features of system behavior. This formulation substantially reduces the effective dimensionality of the model, making it comparable in parameter richness to a standard Lotka–Volterra system with four species (see also SI E).

Extending the framework to larger communities could provide further analytical and numerical insight into invasion dynamics in multi-species communities (see SI F). However, as the number of host species (*M*) and microbial species (*N*) increases, the number of interactions grows proportionally to $(M+N)^2$. This scaling renders both analysis and empirical application increasingly challenging. In practice, estimating the interaction coefficients $(Q_p,Q_m)$ and competition coefficients ($C_p,C_m$) for large systems requires either substantial data or strong simplifying assumptions, as exhaustive information is rarely obtainable from natural systems or controlled experiments. One promising alternative is to treat interaction parameters as random variables drawn from distributions defined by a limited set of statistical features, an approach developed in recent work on large Lotka–-Volterra systems (Akjouj et al. 2024). Such strategies may offer a path forward for extending our framework while maintaining tractability, balancing realism with generality.

## Conclusion

It has been recognized that novel host-symbiont associations can both facilitate invasion and enhance the resilience of native communities. Here, we show that these general patterns can arise from a set of distinct ecological mechanisms, which we formalize into a framework for understanding invasion dynamics more broadly. Our model incorporates the formation of new associations between native and non-native hosts and symbionts, encompassing both mutualistic and parasitic interactions. Results reveal additional, previously overlooked theoretical pathways of invasion, supporting both theoretical and empirical exploration of these dynamics.

## Supplementary Information

Below is the link to the electronic supplementary material.

Supplementary file 1 (pdf 1994 KB)

## Data Availability

The datasets generated or analyzed during the study are available in this published article. All the code developed for the manuscript is available on GitHub. The code is novel and original, and has been developed by author Maria M. Martignoni (2023) and can be found at https://github.com/nanomaria/microbiallymediatedinvasion. Interactive reproduction and re-parametrization of results can also be done through the modelRxiv platform (https://modelrxiv.org/model/YfndNX).
